# Characterizing the Phylogenetic Tree Community Structure of a Protected Tropical Rain Forest Area in Cameroon

**DOI:** 10.1371/journal.pone.0098920

**Published:** 2014-06-17

**Authors:** Stéphanie Manel, Thomas L. P. Couvreur, François Munoz, Pierre Couteron, Olivier J. Hardy, Bonaventure Sonké

**Affiliations:** 1 Aix Marseille Université, IRD, LPED UMR 151, Marseille, France; 2 Centre de coopération internationale en recherche agronomique pour le développement, UMR AMAP, Montpellier, France; 3 Institut de Recherche pour le Développement, UMR DIADE, Montpellier, France; 4 Département des Sciences Biologiques, Laboratoire de Botanique Systématique et d’Ecologie, Université de Yaoundé I, Ecole Normale Supérieure, Yaoundé, Cameroon; 5 Université Montpellier 2, UMR AMAP, Montpellier, France; 6 Institut de Recherche Pour le Développement, UMR AMAP, Montpellier, France; 7 Université Libre de Bruxelles, Faculté des Sciences, Evolutionary Biology and Ecology Brussels, Belgium; University of Oxford, United Kingdom

## Abstract

Tropical rain forests, the richest terrestrial ecosystems in biodiversity on Earth are highly threatened by global changes. This paper aims to infer the mechanisms governing species tree assemblages by characterizing the phylogenetic structure of a tropical rain forest in a protected area of the Congo Basin, the Dja Faunal Reserve (Cameroon). We re-analyzed a dataset of 11538 individuals belonging to 372 taxa found along nine transects spanning five habitat types. We generated a dated phylogenetic tree including all sampled taxa to partition the phylogenetic diversity of the nine transects into alpha and beta components at the level of the transects and of the habitat types. The variation in phylogenetic composition among transects did not deviate from a random pattern at the scale of the Dja Faunal Reserve, probably due to a common history and weak environmental variation across the park. This lack of phylogenetic structure combined with an isolation-by-distance pattern of taxonomic diversity suggests that neutral dispersal limitation is a major driver of community assembly in the Dja. To assess any lack of sensitivity to the variation in habitat types, we restricted the analyses of transects to the terra firme primary forest and found results consistent with those of the whole dataset at the level of the transects. Additionally to previous analyses, we detected a weak but significant phylogenetic turnover among habitat types, suggesting that species sort in varying environments, even though it is not predominating on the overall phylogenetic structure. Finer analyses of clades indicated a signal of clustering for species from the Annonaceae family, while species from the Apocynaceae family indicated overdispersion. These results can contribute to the conservation of the park by improving our understanding of the processes dictating community assembly in these hyperdiverse but threatened regions of the world.

## Introduction

Tropical rain forests are the most biodiverse terrestrial ecosystems on Earth, containing over 50% of known terrestrial biodiversity packed in just 7-10% of the Earth’s surface [1], [2]. The level of biodiversity is remarkable both locally (alpha diversity) and in terms of variation in space (beta diversity; e.g. [3], [4]). Despite the importance of rain forests for terrestrial biodiversity, the drivers of diversity gradients within and between the world’s main rain forest areas remain poorly understood [5], [6]. In addition, large areas of rain forests, among which the central African block, are poorly explored by scientists, giving a fragmentary view of spatial diversity patterns, even for well-studied organisms such as plants [7].

In the last century, rain forests have been overexploited in many parts of the world leading to their alteration, fragmentation, and in some areas, complete destruction [7]. The consequences of these changes include biodiversity loss and increased atmospheric carbon dioxide concentration resulting in climate change, due to the conversion of high-carbon storage forest to low-carbon storage agriculture [8]. As a consequence, there is an urgent need to better understand the processes that sustain the biological diversity in tropical rain forests [9].

Biodiversity is classically assessed at species level (e.g. [10]), from the observation of the presence/absence of species (i.e. species occurrence) or species abundance in transects or ecological plot surveys (e.g. [11]). However, biodiversity assessments based on species counts and their relative abundance statistics provide little information regarding the functional diversity of the ecosystem under study, since they do not acknowledge the variation in their ecological niches [12], [13], [14], [15]. Estimation of phylogenetic or functional diversity in addition to species diversity has been recognized as improving our understanding of the niche-based processes leading to the observed patterns of present day biodiversity [15], [16]. Those estimations also help to better conserve phylogenetic diversity (e.g. [14], [15]). In this context, phylogenetic relatedness is classically considered as a proxy of functional relatedness, because the closer the species are in the phylogeny, the more likely they have inherited similar traits from a common ancestor. As a consequence the consideration of phylogenetic diversity informs on ecosystem functioning and adaptability. This ‘ecophylogenetic’ approach is therefore a relevant basis for conservation purposes [17].

Recently, progress in phylogeny reconstruction either from DNA sequences or from existing resources has accelerated theoretical and methodological advances in ecophylogenetics [18]. The number of studies on phylogenetic alpha and beta diversity in tropical tree communities has increased in the last two or three years [9]. Those studies have focused on partitioning diversity into spatial and environmental components (e.g. [19], [20], [21]) or by comparing the alpha and beta components of phylogenetic diversity (e.g. [18], [20]).

Recent studies carried out on tropical rain forest trees reported that (i) phylogenetic turnover (i.e. a spatial turnover of the dominance of clades) is associated with habitat or environmental differentiation [19], [20], [22], [23]; (ii) functional traits [22] and climate niche proxies [23] usually display a significant, although sometimes weak, phylogenetic structure; (iii) phylogenetic turnover parallels functional turnover, although with a weaker strength [22]. It is noteworthy that most of these studies were carried out in regions with substantial climatic and/or edaphic gradients, so that environmental filtering effects favoring functional and phylogenetic clustering may predominate over competitive exclusion effects that might lead to functional and phylogentic overdispersion (but see [24]).

In this paper we will focus on a region located in the margin of the Congo Basin which is home to the second largest tropical rain forest after the Amazon basin, with a high level of species diversity and endemism [25]. In recent years, several countries have created national parks in an effort to conserve rain forest biodiversity (e.g. Gabon). The Dja Faunal Reserve (DFR) is a UNESCO world heritage reserve of 526,000 ha located 250 kilometers south east from the Cameroon capital Yaoundé. The reserve was established in 1950 and is the largest protected rain forest in Cameroon [26]. There is no steep climatic or edaphic macrogeographic gradient across the DFR. However, its topography characterized by half-orange shaped hills generates a heterogeneity of soil hydromorphy, so that distinct edaphic habitats can be recognized. Besides, natural or past human disturbances (gap dynamics; ancient agricultural fields) are recognized locally by affecting the structure of the vegetation. In a previous study, Hardy and Sonké [27] assessed the role of dispersal and niche differentiation in shaping tree species turnover along nine transects covering the DFR. To this end, they quantified the impact of spatial distance and habitat differentiation on the probability that pairs of individuals are conspecific. With the exception of pioneer species, they found a pattern of isolation by distance, i.e. spatial species clustering due to the combined effect of limited dispersal and local ecological drift [10]. Habitat differentiation was also found to be a major determinant of the spatial pattern but had a lower impact than spatial distance per se. These results suggest that in the DRF, the degree of species aggregation might be better determined by dispersal-assembly rules rather than by niche-assembly rules, at least for the common species. Because this study, which was based on taxonomic diversity, did not take into account phylogenetic diversity between species, a reanalysis of the dataset used in Hardy and Sonke [27], but accounting for phylogenetic variation will provide further insights in how niche-based processes constrains the composition of communities [23]. Because macrogeographic environmental gradients are weak across the DFR compared to most previous works carried out at a similar scale in tropical rain forests, patterns of phylogenetic structure might differ.

The main objective of the paper was to investigate the relative importance of niche-based and dispersal-based processes governing tree species assemblage within the Dja Faunal Reserve. We used an ecophylogenetic approach [14], [15] to provide a phylogenetic quantification of biodiversity in this area for better conservation strategies. We wanted to address the question: can we detect phylogenetic or species turnover across the reserve? Specifically, we elaborated our approach to test the following four hypotheses:

(i) If community assembly is dominated by limited dispersal, no phylogenetic structure should be detected between transects, while isolation-by-distance is expected in taxonomic beta diversity.

(ii) If environmental filtering differed among habitat types a pattern of phylogenetic clustering in habitat should be detected. This interpretation assumes phylogenetic niche conservatism of relevant traits [22], [23].

(iii) If competitive exclusion prevents the co-occurrence of related species locally, generating a patchwork distribution of functionally equivalent species, phylogenetic overdispersion might be detected within transects, at least within a habitat type.

(iv) Environmental filtering and competitive exclusion may simultaneously occur and cancel each other out to yield apparent “neutral” patterns.

A critical issue for testing the relative imprint of these processes is to define sampling units that are relevant according to the scale of the processes. Here we considered the nine transects of Hardy and Sonké [27] that provide information on forest tree composition at two levels: among transects and among habitat types.

**Figure 1:**
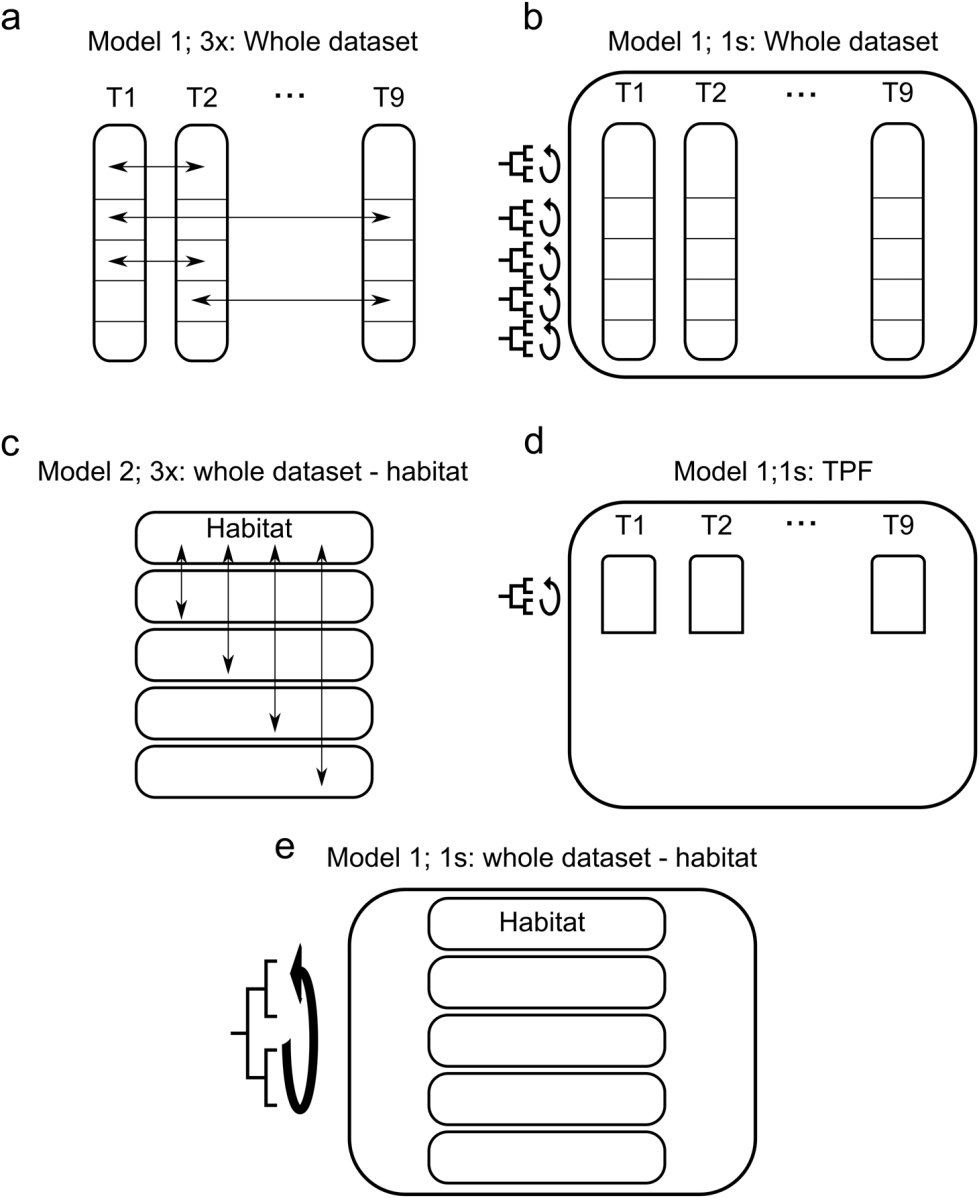
Schema of the 3 types of randomization used to test taxonomic and phylogenetic structure. In model 1-3x, individuals were randomized among transects or species within each habitat type (a). In model 2–3x, individuals or species were randomized among habitats (c). These models of permutation aimed to test for taxonomic turnover using I_ST._ Phylogenetic structure (B_ST_ and Π_ST_) was tested using a model 1 s which randomized the observed species across the tips of the phylogenetic tree (b, d, e). Randomization were respectively done for the whole dataset (a,b), for the habitat data set (c, e) and for TPF only (d).

To address the four hypotheses above, we therefore partitioned taxonomic and phylogenetic diversity within and between transects, as well as within and between habitat types. For this we applied the statistical framework developed by Hardy and Senterre [20] for characterizing and testing the phylogenetic structure of transects and habitats types using appropriate randomization procedures [28] (Table 1, Figure 1).

**Table 1:** Permutation used to test taxonomic and phylogenetic structure in relation with our hypotheses.

Hypothesis	Permutations	Tests
(i) If community assembly is dominated by limited dispersal, no phylogenetic structure should be detected among transects, while isolation-by-distance is expected in taxonomic beta diversity	Model 1-3x Whole dataset - transect. Permutation of individuals between transects within habitat types.	I_ST_
	Model 1 s Whole dataset-transect Permutation of species in phylogeny	B_ST_ - Π_ST_
(i) Isolation-by-distance is expected in taxonomic beta diversity.	Model 2–3x Whole dataset -habitat. Permutations of individuals among habitats	I_ST_
(ii) If environmental filtering differed among habitat types a pattern of phylogenetic clustering between habitats should be detected	Model 1s - Whole dataset-habitat. Permutations of species in phylogeny	B_ST -_ Π_ST_
(iv) Environmental filtering and competitive exclusion may simultaneously occur and cancel out to yield apparently “neutral” patterns		
(iii) If competitive exclusion/niche differentiation prevents the co-occurrence of related species locally, generating a patchwork distribution of functionally equivalent species, phylogenetic overdispersion might be detected within transects, at least within a habitat type	Model 1 s -TPF. Permutations of species in phylogeny within TPF only	B_ST -_ Π_ST_
(iv) Environmental filtering and competitive exclusion may simultaneously occur and cancel out to yield apparently “neutral” patterns		

## Materials and Methods

### 1. Study site and tree communities

**Figure 2:**
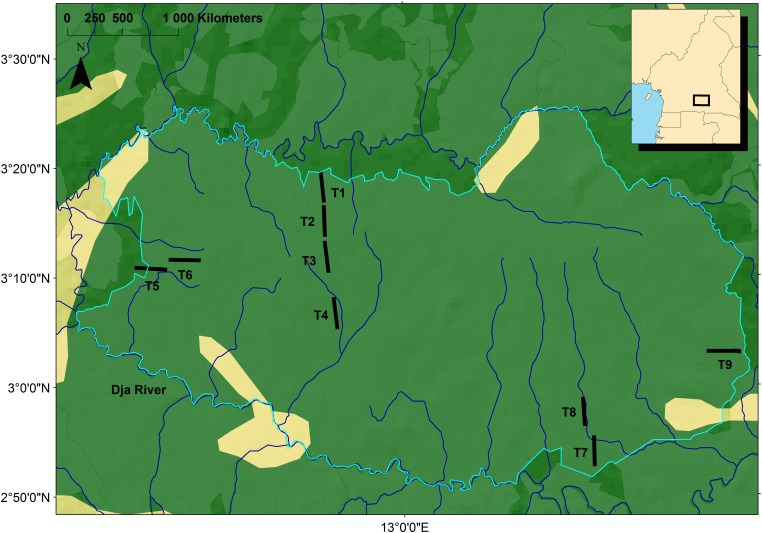
Map of the sampled species with a diameter larger than 10 cm identified in the 5 meter wide first transect. Numbers indicated species richness in each transect.

The Dja Faunal Reserve (DFR) is situated between latitudes 2°50’–3°30’ N and longitudes 12°20’–13°40 E in southeastern Cameroon. About two-thirds of the reserve’s perimeter is demarcated by the Dja River, forming a natural boundary. In this reserve, tree species have been inventoried along nine 5 km long and 5 m wide transects (Figure 2), in which all species with a diameter at breast height bigger than 10 cm were identified and mapped. This dataset (sampling, preliminary taxonomic analysis) is described in Sonké [29] and Sonké and Couvreur [30]. In total 11546 individuals were inventoried belonging to 312 identified species and 60 taxa identified to generic level only (and considered as a morphospecies, 372 total taxa included in the analyses). All nomenclatural criteria regarding species names and families followed Sonké and Couvreur [30]. The vegetation in the reserve has a 30–40 m tall canopy with emergent trees rising up to 60 m [26]. Detailed descriptions of the structure and species composition of the mixed forests can be found in Sonké [31] and Sonké and Couvreur [30].

The nine transects were established across five broad types of forest (i.e. habitat) depending on soil hydromorphy and vegetation structure [32]. Terra firme forests were established on non-hydromorphic soils and subdivided into three successional types, namely (i) terra firme primary forest (74% of total individuals); (ii) secondary forest (8% of total individuals) ; (iii) gaps (4% of total individuals). Conversely, two hydromorphic types were defined as (iv) swamps (11%) and (v) flooded forest (3%).

We considered two sampling levels for subsequent phylogenetic and taxonomic composition analyses. First, we analyzed the variation within and between the 9 transects, such as the comparison of transects represented the largest spatial scale of beta variation. Second, the variation across the five habitats within transects conveyed the imprint of local niche-based processes. All analyses were performed on three datasets: 1) all individuals within each transect ( = whole data - transect); 2) only individuals found in habitat (i) terra firme primary forest  = ("TPF") within each transect; 3) the five different habitats were treated independently of transects (i.e. transects were merged = "habitat") ( = whole data – habitat). This allowed us to test the different assumptions stated in the introduction (Table 1). Our analysis is different than the one of Hardy and Sonké [27] who estimated the probability that two randomly chosen individuals belong to the same species according to the distance separating them (1) on the combined samples of all transects using individual coordinates and then (2) within the three main habitat types.

### 2. Phylogenetic relationships of the DFR tree community

A phylogenetic tree of 372 species was generated in three steps. First, all species were grafted onto a comprehensive phylogenetic tree using the program PHYLOMATIC v3 [33] (http://phylodiversity.net/phylomatic/). The program generated a tree in which the family relationships of the sampled species followed the angiosperm phylogeny APG III [34] version R20120829. We then manually resolved the generic relationships within most of the families based on specific molecular phylogenies (Table 2), using the software Mesquite [35]. Only relationships that were supported with bootstrap values of more than 70% were taken into account. For families where no phylogenetic information was available or for which the published phylogeny did not provide enough insights into the relationships between genera, generic relationships where left unresolved (polytomies). Finally, we used the branch adjustment algorithm BLADJ implemented in Phylocom [36] to scale the branch lengths based on a set of node age estimates from several publications (Table 2). For this part, we first used the dated phylogeny of Wikstrom et al [37] for major nodes. We also used family level dated trees to further constrain certain nodes (Table 2). Intra- and interspecific branch lengths were assumed to be 0 (i.e. relationships between species and within species are unknown and unresolved).

**Table 2:** References to phylogenetic trees and chronograms used to manually resolve relationships and identify calibration points in families with three or more species sampled in this study.

Family	Phylogenetic relationships	Calibration points
angiosperms		[37]
Annonaceae	[52]	[48]
Apocynaceae	[58]	
Bignoniaceae	[59]	
Chrysobalanceae	[60]	
Clusiaceae	[61]	
Combretaceae	[62]	
Euphorbiaceae	[63]	
Leguminosea: Caesalpinoideae	[64];	
Leguminosea: Mimosoideae	[65]	[65]
Malvaceae	[66] and http://www.malvaceae.info	
Meliaceae	[67], [68]	
Myristicaceae	[69]	
Mytaceae	[70]	
Olecaceae	[71]	
Phyllantaceae	[72]	
Rubiaceae	[73]	[73]
Rutaceae	[74]	
Sapindaceae	[75]	[75]

### 3. Species and phylogenetic structure analyses

We used the measures of phylogenetic distinctness and differentiation within and between transects /habitats introduced by Hardy & Senterre [20]. These statistics are based on the additive partitioning of Rao entropy [38], which lead to differentiation coefficients between transects/habitats that are analogous to the coefficients expressing genetic differentiation among populations in population genetics.

Tests of phylogenetic structure can be biased when there is a non-random phylogenetic distribution of species abundance at regional scale (i.e., in the overall dataset) [28]. Therefore, to test if abundant species were randomly distributed across the phylogeny, we first calculated the Abundance Phylogenetic Deviation (APD) statistic [28]. When APD <0, species abundances are overdispersed, whereas when APD >0, species abundances are clustered (abundant species mainly belong to one or a few clades).

We re-estimated taxonomic diversity for phylogenetic analyses since we used a different strategy from the one used in Hardy and Sonké [27]. We calculated the probabilities that two individuals belonged to different species (Simpson-Gini diversity index) within a transect/habitat (D_IS_) and between transects/habitats (D_IT_), as well as the mean phylogenetic distances (based on the divergence time) between individuals (an index of phylogenetic diversity) within transect/habitat (D_PS_), and between transects/habitats (D_PT_). I_ST_ =  (D_IT_-D_IS_)/D_IT_ then expresses the species turnover between transects/habitats. Taxonomic clustering in transects/habitat is expected to be reflected by I_ST_>0, while taxonomic overdispersion should result in I_ST_<0. P_ST_ =  (D_PT_-D_PS_)/D_PT_ expresses the combined effect of species and phylogenetic turnover. However, as it is difficult to interpret, we do not consider this quantity in our interpretations. In addition, we estimated the mean phylogenetic distances between two non-conspecific individuals sampled at local (i.e. within transect/habitat) or regional scale (i.e. between transects/habitats), respectively denoted as D_BS_ and D_BT_, so that B_ST_  =  (D_BT_-D_BS_)/D_BT_ expressed phylogenetic turnover between transects/habitats independently of species turnover [38]. B_ST_ >0 under local phylogenetic clustering while B_ST_ <0 under local phylogenetic overdispersion.

These estimators require abundance data, and rare species are underemphasized, while the distribution of rare species can also bring useful information on species assembly rules. Thus, we used measures of phylogenetic distinctness based on species incidence [20]. Δ_PS_ is defined as the mean phylogenetic distance between distinct species within transects/habitats and Δ_PT_ between transects/habitats (i.e. mean phylogenetic distance between distinct species sampled from two transects/habitats, averaged over all pairs of transects/habitats). Hence a coefficient analogue to B_ST_ is defined, Π_ST_  =  (Δ_PT_-Δ_PS_)/Δ_PT_ which expresses phylogenetic turnover by the gain of phylogenic distance between species occurring in different sites compared with species occurring in the same site. Π_ST_ is equivalent to B_ST_ but neglects species abundances. This coefficient excludes comparisons of a species with itself. All the estimations were performed using the software SPACoDi [39].

### 4. Testing species and phylogenetic structure

To test for species turnover and phylogenetic structure, we used 3 models of randomization (Table 1, Figure 1). In model 1-3x, we randomized individuals among transects or species within each habitat type (Figure 1a). In model 2–3x, we randomized individuals or species among habitats (Figure 1c). These models of permutation aimed to test for taxonomic turnover using I_ST._ The models 3x in which community composition was randomized but not the position of taxa in the phylogeny has been shown to be biased to test the phylogenetic structure [28]. Thus, to test for phylogenetic structure, we used a third permutation model (model 1s, Figure 1 b, d, e). The model 1s randomizes the observed species across the tips of the phylogenetic tree and allowed testing for phylogenetic structure using B_ST_ and Π_ST_ (Table 1).

We undertook 999 permutations for each model, providing 999 estimations of the above differentiation coefficients under those null models. Deviations of observed coefficient from random coefficients were used to test whether I_ST_ = 0, B_ST_ = 0 or Π_ST_  = 0. A significant test for I_ST_ is expected at least under hypothesis (i) between transects or habitats with the model 1–3x. Under hypothesis (ii) (habitat filtering dominates), we expect B_ST_>0 and Π_ST_ >0 with the whole dataset- habitat (model 1s); under hypothesis (iii) (competitive exclusion between related species dominates), we expect B_ST_<0 and Π_ST_ <0 between transects at least within TPF habitat; while no phylogenetic significant tests should be obtained under hypotheses (i, neutral assembly with limited dispersal) and (iv, compensation between ii and iii) (Table 1). Mantel tests were used to test the relations between pairwise taxonomic (I_ST_) or phylogenetic distances (B_ST_ and Π_ST_) and geographic distances among the 9 transects using the R package vegan [40]. A significant test with I_ST_ but not with B_ST_ or Π_ST_ is expected under hypothesis (i).

Finally, to assess the robustness of the results with respect to the taxonomic scale investigated, and possibly assess whether hypothesis (iv, compensatory effects between habitat filtering and competitive exclusion) might hold, partial randomization of the data between transects was performed on certain clades defined as species rich which were arbitrarily defined as families containing 10 or more sampled species. We also looked at Eudicot and Magnoliales clades. For each clade the coefficients described above were calculated under the 1s model (999 randomization of tree tips). This was done by using the spacodi.per.nodes function in the SpacodiR [41].

## Results

### 1. Phylogenetic tree of the DFR

**Figure 3:**
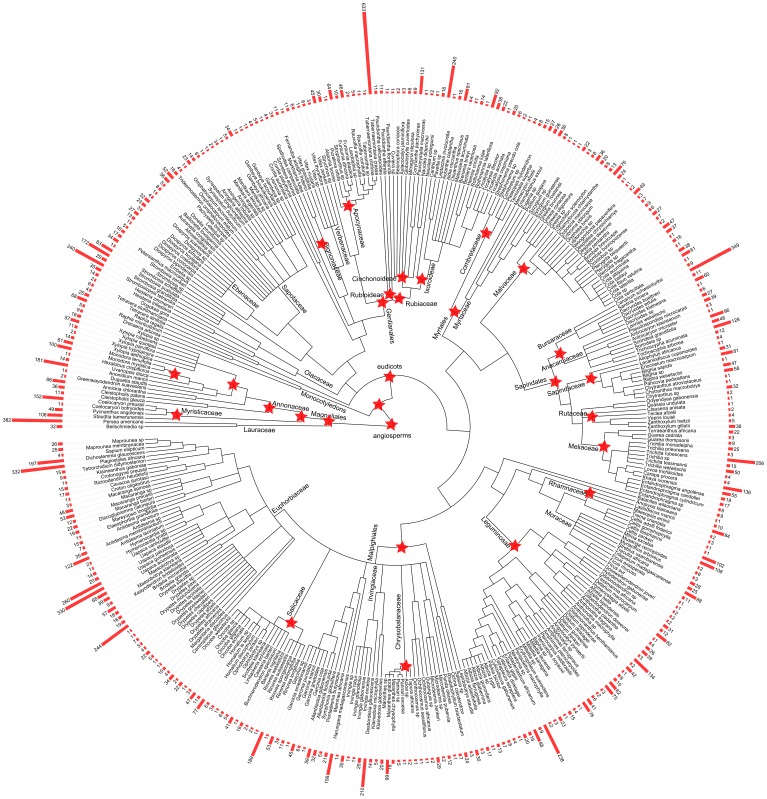
Chronogram of the tree flora of the Dja Faunal Reserve. Branches are proportional to time. Red stars indicate calibration points used in the BALDJ analysis. Species names are indicated in the tips, with their respective abundance information for the whole dataset. Graphic created with using the iTOL web application.

For 17 families, phylogenetic studies allowed the resolution of most relationships between genera (Table 2). A total of 23 calibration points (Table 2) were used to generate the dated phylogenetic tree of the DFR. The tree was produced using the iTOL web application [42], [43] (Figure 3).

### 2. Species and phylogenetic structure analyses

The Abundance Phylogenetic Deviation (APD) estimations were not significant for the 3 datasets (Table 3) indicating that abundant species were randomly distributed across the phylogeny at the scale of the study area.

**Table 3:** Partition of taxonomic and phylogenetic diversity within and between the 9 transects from the Dja Faunal Reserve (11538 individuals belonging to 372 species) for the whole dataset and for TPF only.

Whole dataset	APD = −0.034562	(pval = *0.0750*)	
Coefficients based on	Average within site diversity α	Total diversity (γ = α+β)	Differentiation (β/γ)
Species identity and abundance	D_IS_ = 0.9805	D_IT_ = 0.9871	I_ST_ = 0.0067
Species phylogeny and abundance	D_PS_ = 129.4888	D_PT_ = 130.3916	P_ST_ = 0.0069
	D_BS_ = 132.097	D_BT_ = 132.097	B_ST_ = 0.0002
Species phylogeny and incidence	Δ_PS_ = 129.7548	Δ_PT_ = 129.7781	Π_ST_ = 0.0002
**TPF dataset**	APD = −0.030372	(pval = *0.0880*)	
Coefficients based on	Local diversity α	Total diversity (γ = α+β)	Differentiation (β/γ)
Species identity and abundance	D_PS_ = 129.8353	D_PT_ = 130.8939	P_ST_ = 0.0081
Species phylogeny and abundance	D_PS_ = 129.8353	D_PT_ = 130.8939	P_ST_ = 0.0081
	D_BS_ = 132.64	D_BT_ = 132.672	B_ST_ * = *0.0002
Species phylogeny and incidence	Δ_PS_ = 130.0129	Δ_PT_ = 130.0629	Π_ST_ = 0.0004
**Whole dataset-habitat**	APD = −0.016572	(pvalue = *0.758*)	
Coefficients based on	Local diversity α	Total diversity (γ = α+β)	Differentiation (β/γ)
Species identity and abundance	D_IS_ = 0.9798	D_IT_ = 0.9876	I_ST_ = 0.0079
Species phylogeny and abundance	D_PS_ = 126.8430	D_PT_ = 128.2658	P_ST_ = 0.0111
	D_BS_ = 129.4628	D_BT_ = 129.8765	B_ST_ = 0.0032
Species phylogeny and incidence	Δ_PS_ = 128.9733	Δ_PT_ = 129.0418	Π_ST_ = 0.0005

TPF: terra firme primary forest; APD: mean abundance phylogenetic deviation index.

The probability that two randomly selected individuals belonged to different species (D_IS_) was high for all our 3 datasets (0.9805, 0.9789 and 0.9798 for the whole dataset-transect, TPF and the whole dataset- habitat respectively) (Table 2). The mean divergence time between individuals was D_PS_ = 129.49 million years (Myr), 129.83 Myr and 126.84 Myr respectively for the three datasets. The mean divergence times between species (Δ_PS_) was 129.75 Myr, 130.01 Myr and 128.97 Myr (Table 2). According to these coefficients, most diversity occurred within transect or habitat, the between contribution being always less than or equal to 0.6% for the whole dataset-transect (I_ST_ = 0.0067, B_ST_ = 0.0002, Π_ST_ = 0.0002), 0.7% for TPF (I_ST_ = 0.0079, B_ST_ = 0.0002, Π_ST_ = 0.0004) and 1.1% for the whole dataset-habitat (I_ST_ = 0.0079, B_ST_ = 0.0032, Π_ST_ = 0.0005). When the coefficients were calculated using taxonomic ranks to produce a surrogate of phyletic distances, estimates of phylogenetic distinctness (Δ_PS_ and Δ_PT_) were only slightly different, and estimates of phylogenetic differentiation between transects (Π_ST_) were only slightly affected (Table 3).

**Figure 4:**
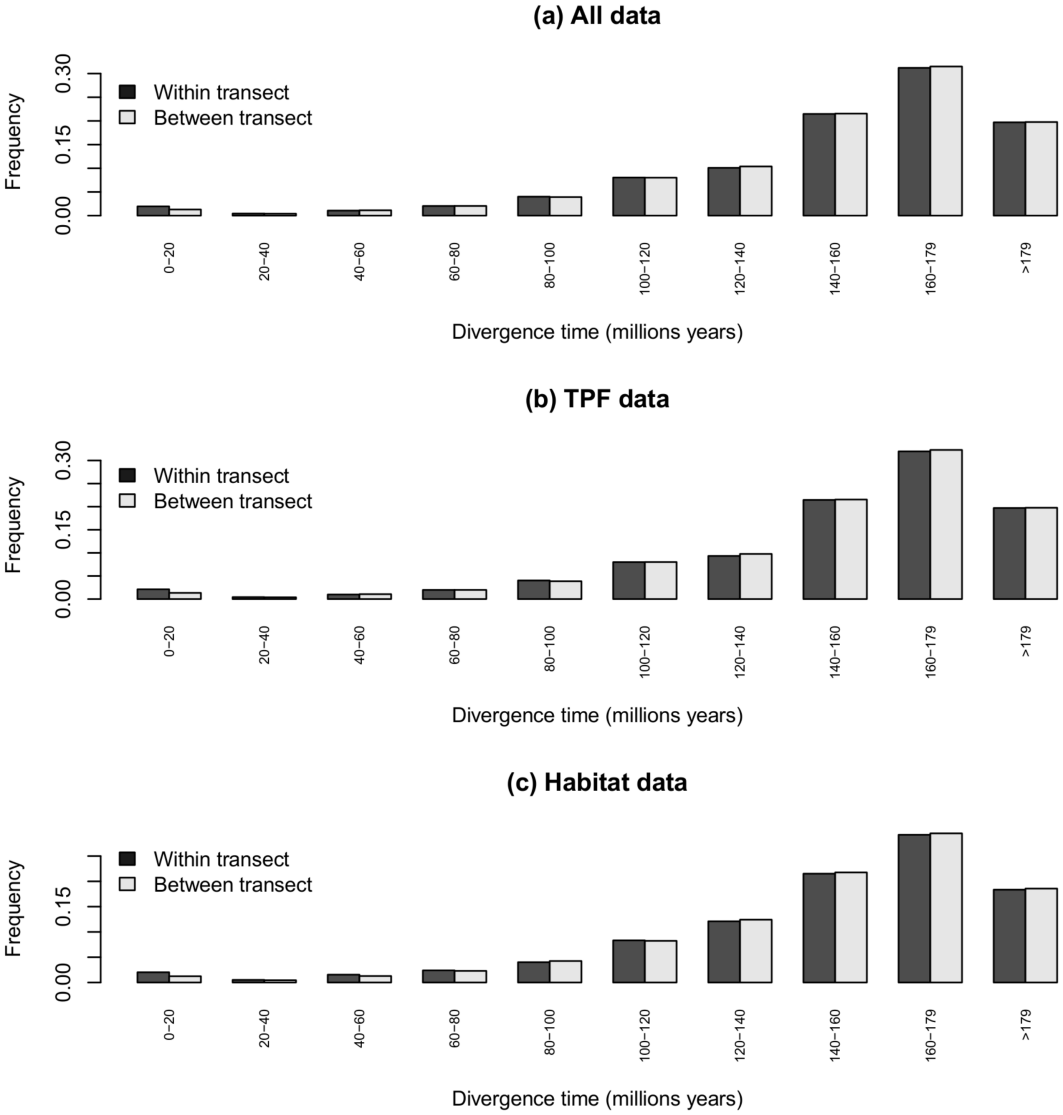
Decomposition within and among plots for Simpson's diversity indices according to the divergence time i.e. frequency distribution of divergence times between individuals from different species for pairs of individuals sampled (a) within transect or between transects for whole data, (b) within transect or between transects for TPF: terra firma primary forest, (c) within habitat or between transects for whole data.

The distribution of divergence times between individuals within a transect and within habitat showed that more than half of the pairs of individuals diverged between 160 and 179 Myr ago (Figure 4).

### 3. Testing species and phylogenetic structure

Whatever the randomization model used (models 1 or 2–3x), all the tests done on taxonomic differentiation were significant (I_ST_ >0), indicating species turnover (Table 4). In the case of model 1–3 x, on the whole dataset at transect level, the test indicated limited dispersal between transects within habitat. The model 2–3x on the whole dataset at habitat level suggested that the turnover is also due to a habitat effect because in the habitat dataset geographic distances are meaningless.

**Table 4:** Testing species and phylogenetic structure within and between transects/habitats.

Dataset	Randomisation	Hypothesis / Results	Interpretation
**Wholedata-transect**	Model 1–3x	H_0_:I_ST_ = 0 pval = 0.000 ***	Limited dispersal between transects within habitat
		H_1_:I_ST_ >0	
	Model 1s	H_0_:B_ST_ = 0 pval = 0.348 NS	No phylogenetic turnover
		H_0_: Π_ST_ = 0 pval = 0.090 NS	
**Whole data-habitat**	Model 2–3x	H_0_:I_ST_ = 0 pval = 0.000 ***	Taxonomic differentiation between habitats: reflect a filtering habitat effect on species
		H_1_:I_ST_ >0	
	Model 1s	H_0_:B_ST_ = 0 pval = 0.05 * (H1: B_ST_>0)	Low phylogenetic turnover for abundant species among habitats
		H_0_:Π_ST_ = 0 pval = 0.225 NS	
**TPF**	Model 1s	H_0_: B_ST_ = 0 pval = 0.364 NS	No phylogenetic turnover
		H_0_: Π_ST_ = 0 pval = 0.064 NS	No phylogenetic turnover

Details of model permutation are given in Table 1. P-values are given after 999 permutations of individuals or species in model of permutation. NS: non significant. Stars indicate the level of the significance.

Concerning tests on phylogenetic structure, we found no significant deviations from random of the observed phylogenetic statistics under model 1 s at the transect level, so that B_ST_ = 0 and Π_ST_ = 0 for the whole dataset-transect and for the TPF dataset. When analyzing the whole dataset at habitat level, the model 1 s still indicated Π_ST_  = 0, but (marginally) significant B_ST_>0 indicating low phylogenetic turnover for abundant species among habitats (Table 4).

Mantel tests indicated that pairwise taxonomic distances (I_ST_, species turnover across space) were significantly correlated to pairwise geographic distances both for the whole dataset-transect as well as for TPF only (r = 0.74, p-val = 9x10^−4^ and r = 0.77, p-val = 0.0013). The relations between pairwise phylogenetic and geographic distances were never significant.

### 4. Variation in phylogenetic structure across the phylogeny

Ten families (sensu AGP III) contained ten or more species [30]. Out of those, only two families exhibited a significant phylogenetic turnover between transects (I_ST_ or P_ST_) under the null model 1 s: Annonaceae (B_ST_  = 0.0009, p-value = 0.04; I_ST_  = 0.0256) and Apocynaceae (B_ST_  = −0.0004, p-value  = 0.019; I_ST_  = 0.0241). Hence, small but significant phylogenetic clustering was identified in Annonaceae (as B_ST_ is positive); whereas overdispersion was detected in Apocynaceae (as B_ST_ is negative). This indicated that Annonaceae species were more related within transects than species taken from different transects, while Apocynaceae species were more related among than within transects. Finally, our results show no significant phylogenetic turnover for most of the species rich families (8 out of 10) indicating a neutral pattern at the transect level, and probably contributing mostly to the global neutral observed pattern.

## Discussion

The present study aimed to better understand the processes underlying taxonomic and phylogenetic diversity and turnover in the Dja Faunal Reserve. We investigated both the alpha and beta diversity components in order to infer mechanisms of local community assembly, as well as the nature of the turnover of species across space, based from the analysis on 9 transects sampled throughout the reserve at both level, transect and habitat type.

### 1. Phylogenetic structure among transects in the Dja Faunal Reserve

The main result of our study is the absence of phylogenetic structure between transects across the DFR when considering the whole dataset (Table 4). This random phylogenetic pattern suggests that competitive exclusion does not prevent the co-occurrence of related species within transects, and there is no strong environment constraint preventing the co-occurrence of distant species [14]. However it does not mean that these processes do not occur, since their entangled effects can lead to not different from random patterns. Our result keeps true when looking just at species found on terra firme primary rain forest (TPF) (Table 4). Finally, the absence of phylogenetic structure within a habitat type is consistent with the hypothesis that processes structuring habitats are dominated by dispersal assembly rules, independently from the species niche attributes or that filtering and competition apparently cancel out.

The existence of a dispersal limitation is further confirmed by the species turnover increasing with spatial distance (isolation by distance) for both datasets at transect levels, while the phylogenetic turnover is insensitive to distance. Using only taxonomic data for the same set of species and with different statistics, Hardy and Sonké [27] also found isolation by distance to be a major driver of community variation in the DFR. This pattern is consistent with a neutral model of community dynamics assuming species equivalence in terms of patterns but not automatically in terms of processes [44]. Previous analyses of beta diversity in tropical forests confirmed that the taxonomic turnover found between forest plots separated by 0.2 to 50 km are consistent with the expectation of the neutral model [10], indicating that dispersal is an important factor in community assemblage rules in these ecosystems. The sampled transects covered most of the delimited DFR area with a maximum distance between sites of 105 km (Figure 2) which justifies interpreting the results at the scale of the whole reserve. As phylogenetic turnover in tropical tree communities is expected to be related to species sorting along an environmental gradient (e.g. [22], [23]), the absence of phylogenetic turnover among transects indicates that, at coarse grain, no environmental variation influenced the variation in forest composition across space. This would agree with the overall homogeneity of environmental conditions found in the DFR [27]. For example, elevation, which has been shown to be an important factor in species composition in tropical rain forests [20], varies between 600 and 700 meters, and does not generate significant environmental variation. Thus even though there is a significant species turnover across the reserve, phylogenetic relatedness remains equivalent at different places across the Dja rejecting our hypothesis (iii) of the presence of competitive exclusion, and confirming our hypothesis (i), that our communities are marked by dispersal assembly rules. However, we still need to be cautious with our conclusions since an alternative explanations of the fact that no phylogenetic sorting was found among transects might actually not have anything to do with community assembly mechanisms, but rather with the resolution of the phylogeny. Because relationships are only resolved to family/genus level, if species belonging to the same genus are functionally distinct and are sorted among sites we would not be able to tell it with this phylogeny.

Even though our dataset contained only 9 transects, which is fairly limiting, we nevertheless have an extensive coverage of the reserve (Figure 2), and have sampled over 11 000 individuals for 372 species or morphospecies. In addition, significant values of I_ST_ and significant Mantel tests between species and geographic distances among transects indicated that spatial variation among species has been captured in the study. I_ST_ values pointed however towards lower values than those observed for more fragmented wet forests such as in the Western Ghats of India, where I_ST_ values were found above 0.027 (computations from the results of [45], against 0.0067 to 0.0079 here (Table 3); I_ST_ is an increasing function of relative community differentiation). The same pattern is observed when considering Π_ST_ (incidence data integrating phylogeny) which is 2–4 10^−4^ in the Dja Reserve against 34 10^−4^ for the dataset in India and 13 10^−4^ in the Panama Canal watershed [23]. This may indicate that differentiation between localities is not very pronounced in the continuous Dja forest.

### 2. Phylogenetic structure among habitat types in the Dja Faunal Reserve

At habitat level, a signal of phylogenetic turnover was barely significant (P-value  = 0.05) with abundance data but not with incidence data (B_ST_>0 and Π_ST_ ∼0). Since the most constraining habitat types are of limited extent, this may explain why there are some significant patterns with abundance data and not with incidence data. This result suggests that species are sorting not just because of limited colonization but also because of environmental variation due to habitat heterogeneity. Indeed, phylogenetic turnover was expected among habitats because some of the environmental gradients distinguishing between habitat types in the reserve, such as water availability and anoxic stresses (flooded or swamp vegetation versus terra firme vegetation), are known to be strong filtering factors influencing tropical forest community structures [19]. This confirms our second hypothesis (ii): environmental filtering differs among habitat types and relevant selected traits could be phylogenetically conserved although this would have to be further tested using traits dataset.

### 3. Variation in phylogenetic structure across the phylogeny

To date few studies have investigated community phylogenetic structure in African rain forests. In a study of 28 one-hectare plots in mature rain forest in Monte Alén National Park (Equatorial Guinea), Hardy and Senterre [20] found a phylogenetic clustering structure which was attributed to adaptations of local species to elevation gradients. They also found that most of the signal is related to ancient clade subdivisions, with most of the individuals pairs (between and among plots) occurring between 100 and 120 Myr. In our analysis, we find a comparable situation where most subdivisions between individual pairs occur between 140–160 Myr. The difference could be related to the different calibrations used to date of the phylogenetic trees. Hardy and Senterre [20] used ages from Davis et al. [46] while we used the more conservative value of Wikstrom *et al* [37] to constrain the origin of the angiosperms (150 Myr versus 179 Myr) in addition to several other calibrations points based on detailed family-specific dating analyses (see methods). Approaches whereby DNA sequence data is generated for the whole sampling would possibly provide better resolution at shallower nodes and hence better address more recent signals [47]. However the resolution of our tree is good for ancient lineages but poor for recent ones. As a consequence this pattern should be interpreted with care.

Our results indicate that Annonaceae species are more related within transects than species taken from different transects. This result could be real and not just an artifact of phylogenetic resolution as the phylogenetic tree for Annonaceae is well resolved [48], [49]. Clustering of Annonaceae was also found in another study of African phylogenetic structure in Equatorial Guinea [20] in which phylogenetic differentiation was shown to be correlated with elevation. They also indicated that the number of magnoliid (which includes Annonaceae) species per plot was correlated with altitude. Interestingly, the DFR has very little elevation variability [27] and thus the significant phylogenetic differentiation detected in Annonaceae of the Dja would have to result from a different process than altitudinal gradients. Moreover, in contrast to South America, lowland Annonaceae are more or as diverse at mid latitudinal as indicated in a survey of an elevation gradient in Mont Cameroon [50] . On the contrary, we observed an opposite pattern for Apocynaceae since species among transects appear more related than within indicating an overdispersion of phylogenetic pattern. This result might be linked to the fact that the species *Tabernaemontana crassa* (Apocynaceae) is the most abundant species inventoried in the reserve and strongly present in transects [51].The rest the Apocynaceae species are not well represented (1–64 individuals/species) and less well represented across all transects. In both cases, Annonaceae and Apocynaceae have a large number of liana species [52], [53] that have not been inventoried in this study and thus more detailed sampling and tests should be carried out before a link to any evolutionary pattern can be done. However, the more in depth analyses looking at the species rich clade as the Annonaceae indicates that some phylogenetic sorting is occurring among transects and can be detected when more data is available**.**

### 4. Phylogenetic diversity and conservation

A classical measure of phylogenetic diversity (signal) is Faith’s PD [12] which measures the total phylogenetic branch length (i.e. amount) of evolutionary history in the studied community. This measure is equivalent in our work to Δ_PS_ which also does not account for species abundance. However, Δ_PS_ is a measure of phylogenetic distinctness, but has the advantage not to be influenced by species richness [20].

Recent literature has debated the interest of adding phylogenetic diversity evaluation in conservation planning [17], [54], [55]. One general agreement in favor of taking phylogenetic diversity into account is to conserve all components of biodiversity including evolutionary information, and that the explicit consideration of biodiversity as comprising evolving and related lineages would add power and robustness to measures of biodiversity for conservation [17]. Specifically, adding phylogenetic estimation in conservation strategy would result in maximization of the set of species to be conserved [55]. According to our results, most diversity occurred within transect or habitat, the between contribution being always less than or equal to 1.1% (whole dataset-habitat).

## Conclusions

The preservation of tropical rainforest is an ethical, political and practical concern and biodiversity assessment should be a major focus in nature preservation programs [56]. Indeed, faced with high anthropic pressure in tropical forest, the number and extent of protected areas have increased across the tropics [57]. The objective of such protected areas is to conserve a sufficient sample of the world's biodiversity.

Few conservation policies consider phylogenetic diversity as an important component probably because the added value of phylogenetic diversity for nature conservation remains unclear [55] due to a lack of consensus between various measures and a difficulty to interpret the results in terms of conservation perspectives [54], [55]. Here, we detected a random phylogenetic pattern between transects at the scale of the Dja Faunal Reserve, possibly because of a common history and weak environmental variation. We also showed that geographic distance encompassed species turnover. In addition, our phylogenetic based analysis added new results to the previous study of Hardy and Sonké [27] using the same dataset, by detecting a weak but significant phylogenetic turnover signal among habitats reflecting a filtering effect of the habitat. Our results can contribute to the conservation of the park by providing insights into the processes driving community assembly. Notably, the prevalence of patterns compatible with dispersal assembly highlights the need to conservation schemes that allow for sufficiently large conservation areas. Future studies should investigate more plots to be based on a hierarchical sampling plan considering spatial variation within transects in order to better interpret the phylogenetic structure.

## References

[1] Pimm SL, Raven P (2000) Biodiversity - Extinction by numbers. Nature 403: 843–845. 10706267 10.1038/35002708

[2] Gibson L, Lee TM, Koh LP, Brook BW, Gardner TA, et al (2011) Primary forests are irreplaceable for sustaining tropical biodiversity. Nature 478: 378–381. 21918513 10.1038/nature10425

[3] Koleff P, Lennon JJ, Gaston KJ (2003) Are there latitudinal gradients in species turnover? Global Ecology and Biogeography 12: 483–498.

[4] Kraft NJB, Comita LS, Chase JM, Sanders NJ, Swenson NG, et al (2011) Disentangling the drivers of beta diversity along latitudinal and elevational gradients. Science 333: 1755–1758. 21940897 10.1126/science.1208584

[5] Givnish TJ (1999) On the causes of gradients in tropical tree diversity. Journal of Ecology 87: 193–210.

[6] Parmentier I, Malhi Y, Senterre B, Whittaker RJ, Alonso A, et al (2007) The odd man out? Might climate explain the lower tree alpha-diversity of African rain forests relative to Amazonian rain forests? Journal of Ecology 95: 1058–1071.

[7] Burgess N, Kuper W, Mutke J, Brown J, Westaway S, et al (2005) Major gaps in the distribution of protected areas for threatened and narrow range Afrotropical plants. Biodiversity and Conservation 14: 1877–1894.

[8] Lewis SL (2006) Tropical forests and the changing earth system. Philosophical Transactions of the Royal Society B-Biological Sciences 361: 195–210. 10.1098/rstb.2005.1711 PMC1626535 16553317

[9] Swenson NG (2013) The assembly of tropical tree communities the advances and shortcomings of phylogenetic and functional trait analyses. Ecography 36: 264–276.

[10] Condit R, Pitman N, Leigh EG, Chave J, Terborgh J, et al (2002) Beta-diversity in tropical forest trees. Science 295: 666–669. 11809969 10.1126/science.1066854

[11] Gentry AH (1988) Tree species richness of upper amazonian forests. Proceedings of the National Academy of Sciences of the United States of America 85: 156–159. 16578826 10.1073/pnas.85.1.156 PMC279502

[12] Faith DP (1992) Conservation evaluation and hylogenetic diversity Biological Conservation. 61: 1–10.

[13] Swenson NG, Stegen JC, Davies SJ, Erickson DL, Forero-Montana J, et al (2012) Temporal turnover in the composition of tropical tree communities: functional determinism and phylogenetic stochasticity. Ecology 93: 490–499. 22624204 10.1890/11-1180.1

[14] Webb CO, Ackerly DD, McPeek MA, Donoghue MJ (2002) Phylogenies and community ecology. Annual Review of Ecology and Systematics 33: 475–505.

[15] Mouquet N, Devictor V, Meynard CN, Munoz F, Bersier LF, et al (2012) Ecophylogenetics: advances and perspectives. Biological Reviews 87: 769–785. 22432924 10.1111/j.1469-185X.2012.00224.x

[16] McGill BJ, Enquist B, Weiher E, Westoby M (2006) Rebuilding community ecology from functional traits. Trends in Ecology and Evolution 14: 178–185. 10.1016/j.tree.2006.02.002 16701083

[17] Diniz-Filho JAF, Loyola RD, Raia P, Mooers AO, Bini LM (2013) Darwinian shortfalls in biodiversity conservation. Trends in ecology & evolution (Personal edition). 10.1016/j.tree.2013.09.003 24091208

[18] Swenson NG, Erickson DL, Mi XC, Bourg NA, Forero-Montana J, et al (2012) Phylogenetic and functional alpha and beta diversity in temperate and tropical tree communities. Ecology 93: S112–S125. 10.1890/11-1180.1 22624204

[19] Fine PVA, Kembel SW (2011) Phylogenetic community structure and phylogenetic turnover across space and edaphic gradients in western Amazonian tree communities. Ecography 34: 552–565.

[20] Hardy OJ, Senterre B (2007) Characterizing the phylogenetic structure of communities by an additive partitioning of phylogenetic diversity. Journal of Ecology 95: 493–506.

[21] Swenson NG, Anglada-Cordero P, Barone JA (2011) Deterministic tropical tree community turnover: evidence from patterns of functional beta diversity along an elevational gradient. Proceedings of the Royal Society B-Biological Sciences 278: 877–884. 10.1098/rspb.2010.1369 PMC3049044 20861048

[22] Baraloto C, Hardy OJ, Paine CET, Dexter KG, Cruaud C, et al (2012) Using functional traits and phylogenetic trees to examine the assembly of tropical tree communities. Journal of Ecology 100: 690–701.

[23] Hardy OJ, Couteron P, Munoz F, Ramesh BR, Pelissier R (2012) Phylogenetic turnover in tropical tree communities: impact of environmental filtering, biogeography and mesoclimatic niche conservatism. Global Ecology and Biogeography 21: 1007–1016.

[24] Mayfield MM, Levine JM (2010) Opposing effects of competitive exclusion on the phylogenetic structure of communities. Ecology Letters 13: 1085–1093. 20576030 10.1111/j.1461-0248.2010.01509.x

[25] White F (1983) The vegetation of Africa. A descriptive memoir to accompagn the UNESCO/AEFTFAT/UNSO vegatation map of Africa. Paris, Copedith.

[26] McGinley M (2008) Dja Faunal Reserve. In Cleveland, C.J. (ed) Encyclopedia of Earth. United Nations Environment Programme–World Conservation Monitoring Centre. http://www/eoearth.org/article/Dja_Faunal_Reserve,_Cameroon.

[27] Hardy OJ, Sonke B (2004) Spatial pattern analysis of tree species distribution in a tropical rain forest of Cameroon: assessing the role of limited dispersal and niche differentiation. Forest Ecology and Management 197: 191–202.

[28] Hardy OJ (2008) Testing the spatial phylogenetic structure of local communities: statistical performances of different null models and test statistics on a locally neutral community. Journal of Ecology 96: 914–926.

[29] Sonké B (1998) Etudes floristiques et structurales des forêts de la Réserve de Faune du Dja (Cameoun). Thèse de Ph. D. Recherche, Université Libre deBruxelles: 129–130.

[30] Sonké B, Couvreur TLP (2014) Tree diversity of the Dja Faunal Reserve, South Caleroon. Biodiversity Data Journal 2: e1049 DOI:10.3897/BDJ.2.e1049 10.3897/BDJ.2.e1049 PMC4030213 24855441

[31] Sonké B (2004) Forêts de la réserve du Dja (Cameroun). Etudes floristiques et structurales. Scripta Botanica Belgica 32: 1–144.

[32] Lebrun J, Gilbert GS (1954) Une classification éccologique des forêts du Congo. Publication de l'Institut National pour l'étude AGronomique du Congo Belge 63: 9–89.

[33] Webb CO, Donoghue MJ (2005) Phylomatic: tree assembly for applied phylogenetics. Molecular Ecology Notes 5: 181–183.

[34] TAP G (2009) An update of the Angiosperm Phylogeny Group classification for the orders and families of flowering plants: APG III. Botanical Journal of the Linnean Society 161: 105–121.

[35] Maddison W, Maddison D (2009) Mesquite: A modular system for evolutionary analysis. version 2.7. http://mesquiteproject.org.

[36] Webb CO, Ackerly DD, Kembel SW (2008) Phylocom: software for the analysis of phylogenetic community structure and trait evolution. Bioinformatics 24: 2098–2100. 18678590 10.1093/bioinformatics/btn358

[37] Wikstrom N, Savolainen V, Chase MW (2001) Evolution of the angiosperms: calibrating the family tree. Proceedings of the Royal Society B-Biological Sciences 268: 2211–2220. 10.1098/rspb.2001.1782 PMC1088868 11674868

[38] Hardy OJ, Jost L (2008) Interpreting and estimating measures of community phylogenetic structuring. Journal of Ecology 96: 849–852.

[39] Hardy OJ (2010) SPACoDi 0.10: a program dor spatial & phylogenetic analysis of community diversity. AVailable at http://ebe.ulb.ac.be/ebe/Software.html.

[40] Team RDC (2012) R: A Language and Environment for Statistical Computing. R Foundation for Statistical Computing.

[41] Eastman JM, Paine CET, Hardy OJ (2011) spacodiR: structuring of phylogenetic diversity in ecological communities. Bioinformatics 27: 2437–2438. 21737436 10.1093/bioinformatics/btr403

[42] Letunic I, Bork P (2007) Interactive Tree Of Life (iTOL): an online tool for phylogenetic tree display and annotation. Bioinformatics 23: 127–128. 17050570 10.1093/bioinformatics/btl529

[43] Letunic I, Bork P (2011) Interactive Tree of Life v2: online annotation and display of phylogenetic trees made easy. Nucleic Acids Research 39: W475–W478. 21470960 10.1093/nar/gkr201 PMC3125724

[44] Hubbell S (2001) A unified neutral theory of biodiversity and Biogeography. Princeton University Press, Princeton.

[45] Munoz F, Couteron P, Ramesh BR (2008) Beta diversity in spatially implicit neutral models: A new way to assess species migration. American Naturalist 172: 116–127. 10.1086/587842 18533823

[46] Davies TJ, Barraclough TG, Chase MW, Soltis PS, Soltis DE, et al (2004) Darwin's abominable mystery: Insights from a supertree of the angiosperms. Proceedings of the National Academy of Sciences of the United States of America 101: 1904–1909. 14766971 10.1073/pnas.0308127100 PMC357025

[47] Kress WJ, Erickson DL, Swenson NG, Thompson J, Uriarte M, et al. (2010) Advances in the Use of DNA Barcodes to Build a Community Phylogeny for Tropical Trees in a Puerto Rican Forest Dynamics Plot. PLoS ONE 5. 10.1371/journal.pone.0015409 PMC2976767 21085700

[48] Couvreur TLP, Pirie MD, Chatrou LW, Saunders RMK, Su YCF, et al (2011) Early evolutionary history of the flowering plant family Annonaceae: steady diversification and boreotropical geodispersal. Journal of Biogeography 38: 664–680.

[49] Chatrou LW, Pirie MD, Erkens RHJ, Couvreur TLP, Neubig KM, et al (2012) A new subfamilial and tribal classification of the pantropical flowering plant family Annonaceae informed by molecular phylogenetics. Botanical Journal of the Linnean Society 169: 5–40.

[50] Bele MY, Focho DA (2011) Inventory and distribution of the Annonaceae along elevation gradient on Mount Cameroon. Journal of Horticulture and Forestry 10: 307–319.

[51] Sonké B, Couvreur TLP (2014) Tree diversity of the Dja Faunal Reserve, South Cameroon. Biodiversity Data Journal 2: e1049. 10.3897/BDJ.2.e1049 PMC4030213 24855441

[52] Chatrou L, Pirie M, Erkens R, Couvreur T, Neubig KM, et al (2012) A new subfamilial and tribal classification of the pantropical flowering plant family Annonaceae informed by molecular phylogenetics. Botanical Journal of the Linnean Society 169: 5–40.

[53] Lahaye R, Civeyrel L, Speck T, Rowe N (2005) Evolution of shrub-like growth forms in the lianoid subfamily Secamonoideae (Apocynaceae s.l.) of Madagascar: phylogeny, biomechanics, and development. American Journal of Botany 92: 1381–1396. 21646158 10.3732/ajb.92.8.1381

[54] Rolland J, Cadotte MW, Davies J, Devictor V, Lavergne S, et al (2012) Using phylogenies in conservation: new perspectives. Biology Letters 8: 692–694. 22130171 10.1098/rsbl.2011.1024 PMC3440956

[55] Winter M, devictor V, Schweiger O (2013) Phylogenetic diversity and nature conservation : where are we? Trends in Ecology & Evolution 28: 199–204. 23218499 10.1016/j.tree.2012.10.015

[56] Chave J, Wiegand K, Levin S (2002) Spatial and biological aspects of reserve design. Environmental Modeling & Assessment 7: 115–122.

[57] Jenkins CN, Joppa L (2009) Expansion of the global terrestrial protected area system. Biological Conservation 142: 2166–2174.

[58] Potgieter K, Albert VA (2001) Phylogenetic Relationships within Apocynaceae s.l. Based on trnL Intron and trnL-F Spacer Sequences and Propagule Characters. Annals of the Missouri Botanical Garden 88: 523–549.

[59] Olmstead RG, Zjhra ML, Lohmann LG, Grose SO, Eckert AJ (2009) A molecular phylogeny and classification of Bignoniaceae. American Journal of Botany 96: 1731–1743. 21622359 10.3732/ajb.0900004

[60] Yakandawala D, Morton CM, Prance GT (2010) Phylogenetic Relationships of the Chrysobalanaceae Inferred from Chloroplast, Nuclear, and Morphological Data1. Annals of the Missouri Botanical Garden 97: 259–281.

[61] Gustafsson MHG, Bittrich V, Stevens PF (2002) Phylogeny of Clusiaceae Based on rbcL sequences. International Journal of Plant Sciences 163: 1045–1054.

[62] Tan F, Shi S, Zhong Y, Gong X, Wang Y (2002) Phylogenetic relationships of Combretoideae (Combretaceae) inferred from plastid, nuclear gene and spacer sequences. Journal of Plant Research 115: 475–481. 12579451 10.1007/s10265-002-0059-1

[63] Wurdack KJ, Hoffmann P, Chase MW (2005) Molecular phylogenetic analysis of uniovulate Euphorbiaceae (Euphorbiaceae sensu stricto) using plastid RBCL and TRNL-F DNA sequences. American Journal of Botany 92: 1397–1420. 21646159 10.3732/ajb.92.8.1397

[64] Bruneau A, Forest F, Herendeen PS, Klitgaard BB, Lewis GP (2001) Phylogenetic Relationships in the Caesalpinioideae (Leguminosae) as inferred from chloroplast trnL intron sequences. Systematic Botany 26: 487–514.

[65] Lavin M, Herendeen PS, Wojciechowski MF (2005) Evolutionary rates analysis of leguminosae implicates a rapid diversification of lineages during the tertiary. Systematic Biology 54: 575–594. 16085576 10.1080/10635150590947131

[66] Alverson WS, Whitlock BA, Nyffeler R, Bayer C, Baum DA (1999) Phylogeny of the core Malvales: evidence from ndhF sequence data. American Journal of Botany 86: 1474–1486. 10523287

[67] Muellner AN, Pennington TD, Koecke AV, Renner SS (2010) Biogeography of Cedrela (Meliaceae, Sapindales) in central and south America. American Journal of Botany 97: 511–518. 21622412 10.3732/ajb.0900229

[68] Muellner AN, Samuel R, Johnson SA, Cheek M, Pennington TD, et al (2003) Molecular phylogenetics of Meliaceae (Sapindales) based on nuclear and plastid DNA sequences. American Journal of Botany 90: 471–480. 21659140 10.3732/ajb.90.3.471

[69] Sauquet H, Doyle JA, Scharaschkin T, Borsch T, Hilu KW, et al (2003) Phylogenetic analysis of Magnoliales and Myristicaceae based on multiple data sets: implications for character evolution. Botanical Journal of the Linnean Society 142: 125–186.

[70] Wilson PG, O’Brien MM, Heslewood MM, Quinn CJ (2005) Relationships within Myrtaceae sensu lato based on a *mat* K phylogeny. Plant Systematics and Evolution 251: 3–19.

[71] Malécot V, Nickrent DL (2008) Molecular Phylogenetic Relationships of Olacaceae and Related Santalales. Systematic Botany 33: 97–106.

[72] Wurdack KJ, Hoffmann P, Samuel R, de Bruijn A, van der Bank M, et al (2004) Molecular phylogenetic analysis of Phyllanthaceae (Phyllanthoideae pro parte, Euphorbiaceae sensu lato) using plastid RBCL DNA sequences. American Journal of Botany 91: 1882–1900. 21652335 10.3732/ajb.91.11.1882

[73] Bremer B, Eriksson T (2009) Time tree of Rubiaceae: phylogeny and dating the family, subfamilies, and tribes. International Journal of Plant Sciences 170: 766–793.

[74] Groppo M, Pirani JR, Salatino MLF, Blanco SR, Kallunki JA (2008) Phylogeny of Rutaceae based on twononcoding regions from cpDNA. American Journal of Botany 95: 985–1005. 21632420 10.3732/ajb.2007313

[75] Buerki S, Forest F, Acevedo-Rodríguez P, Callmander MW, Nylander JAA, et al (2009) Plastid and nuclear DNA markers reveal intricate relationships at subfamilial and tribal levels in the soapberry family (Sapindaceae). Molecular Phylogenetics and Evolution 51: 238–258. 19405193 10.1016/j.ympev.2009.01.012

